# Low birth weight and intrauterine growth restriction and their relationship with handgrip strength in adolescents

**DOI:** 10.1590/1984-0462/2026/44/2025226

**Published:** 2026-08-28

**Authors:** Brena Cristina Batista Barros, Juliana Ramos Carneiro, Vanessa de Oliveira Martins, Rosângela Fernandes Lucena Batista, Vanda Maria Ferreira Simões, Antônio Augusto Moura da Silva, Liliana Yanet Gómez Aristizábal, Susana Cararo Confortin

**Affiliations:** a Universidade Federal do Maranhão, São Luís, MA, Brazil.; b Universidad CES, Medellín, Antioquia, Colômbia.; c Universidade do Extremo Sul Catarinense, Criciúma, SC, Brazil.

**Keywords:** Fetal growth retardation, Low birth weight, Hand grip strength, Adolescent, Retardo do crescimento fetal, Baixo peso ao nascer, Força de preensão manual, Adolescente

## Abstract

**Objective:**

To analyze the association between low birth weight (LBW) and intrauterine growth restriction (IUGR) with handgrip strength (HGS) in adolescents.

**Methods:**

Birth cohort study from São Luís, Maranhão, Brazil, conducted between 1997 and 1998. The Jamar Plus+ dynamometer was used to measure HGS during the second follow-up. Birth weight was assessed from information from the participants’ hospital records. LBW was defined as a birth weight <2500 g, while IUGR was defined as a Kramer’s index <0.85. The associations of LBW and IUGR with HGS were estimated using linear regression models. The directed acyclic graph (DAG) was used to identify the variables for adjustment.

**Results:**

We evaluated 544 individuals who participated in the baseline and second follow-up, and 279 who participated in the first and second follow-ups. In crude and adjusted analyses, LBW was not associated with HGS. However, in the crude analysis, male adolescents with IUGR had lower HGS (β: -4.17; 95%CI -7.67; -0.66) compared with those without IUGR. In the adjusted analysis, the association was maintained, with male adolescents with IUGR showing a reduction of 4.36 kgf (β -4.36; 95%CI -7.88; -0.85) in HGS compared with those without IUGR. There was no association between IUGR and HGS in females.

**Conclusions:**

LBW was not associated with HGS. However, male adolescents with IUGR had lower HGS.

## RESUMO

**Objetivo:**

Analisar a associação entre o baixo peso ao nascer (BPN) e a restrição do crescimento intrauterino (RCIU) com a força de preensão manual (FPM) em adolescentes.

**Métodos:**

Estudo de coorte de nascimento de São Luís, Maranhão, Brasil, realizado entre 1997 e 1998. O dinamômetro Jamar Plus+ foi utilizado para aferir a FPM no segundo acompanhamento. O peso ao nascer foi obtido com base em informações dos prontuários hospitalares dos indivíduos participantes. BPN foi definido como peso ao nascer <2500 g, enquanto RCIU foi definida pelo índice de Kramer <0,85. As associações entre BPN e RCIU com a FPM foram estimadas por modelos de regressão linear. Utilizou-se o gráfico acíclico direcionado (DAG) para identificar as variáveis para ajuste.

**Resultados:**

Foram avaliados 544 indivíduos que participaram da linha de base e do segundo seguimento; e 279 indivíduos que participaram do primeiro e do segundo seguimento. Na análise bruta e ajustada, BPN não foi associado à FPM. Entretanto, na análise bruta, adolescentes do sexo masculino que tiveram RCIU apresentaram menor FPM (β -4,17; IC95% -7,67; -0,66) do que aqueles sem RCIU. Na análise ajustada, a associação se manteve, e os adolescentes do sexo masculino com RCIU apresentaram redução de 4,36 kgf (β -4,36; IC95% -7,88; -0,85) na FPM quando comparados aos sem RCIU. Não houve associação entre a RCIU e a FPM no sexo feminino.

**Conclusões:**

BPN não foi associado à FPM, porém adolescentes do sexo masculino com RCIU apresentaram resultados inferiores de FPM.

## INTRODUCTION

Handgrip strength (HGS) has been useful for assessing nutritional and health statuses,^1,2^ serving as an indicator of muscle strength,^3^ a marker of future physical strength, a predictor of disease risk in adulthood,^4,5^ and a prognostic indicator of adverse outcomes.^3,6^ Reduced muscle strength is linked to sarcopenia, chronic inflammation, and insulin resistance^4,7^ — factors that increase cardiovascular risk and mortality.

Currently, there is growing interest in understanding the determinants of HGS during adolescence. Factors such as age,^7^ sex, nutritional status, body composition, as well as birth weight and intrauterine growth restriction (IUGR) are associated with this strength in the early stages of life.^8,9,10^ Low birth weight (LBW) has been systematically associated with HGS.^6^ Studies have shown that extreme birth weights, both high and low, are related to future nutritional status, predisposing individuals to excess body weight, reduced muscle strength, and lower physical fitness in young adults.^11,12^ One possible mechanism linking LBW to reduced muscle strength involves long-term alterations in muscle histology and morphology, due to an adverse intrauterine environment.^13^ Individuals born with LBW have altered skeletal muscle fiber composition and structure, which may compromise later muscle function.^13^

Another factor associated with lower HGS is IUGR, since the presence of stress during pregnancy increases the risk of fetal growth restriction and the development of health problems known as “fetal programming,” which is likely responsible for lower muscle strength.^9^ Changes such as IUGR lead to postnatal modifications that persist into adulthood.^11^ Evidence from human and animal studies indicates that IUGR leads to structural and functional alterations in brain development that may underlie later motor impairments, including reduced strength and fine motor performance.^14^ IUGR has been associated with reduced total and cortical brain volumes, decreased neuronal cell numbers, myelination deficits, and impaired connectivity, attributable to abnormal neuronal migration and reduced dendritic processes. These abnormalities can result in dysfunctions in motor, cognitive, and behavioral skills.^14^

Although there is research evaluating the isolated effect of both LBW and IUGR on HGS, studies assessing the influence of both variables on HGS are scarce.^10^ In addition, previous studies have generally not addressed the distinct causal structures underlying these exposures. LBW and IUGR are related but conceptually distinct indicators of fetal development: LBW reflects the absolute weight at delivery, whereas IUGR reflects impaired fetal growth relative to gestational age.^14^ Because these indicators capture different dimensions of prenatal development, the causal pathways and potential confounding structures underlying their associations with later muscle strength may differ. Evaluating them within the same cohort allows a more consistent assessment of their associations with adolescent muscle strength. Therefore, the objective of this study is to analyze the associations of LBW and IUGR with HGS in adolescents using separate regression models, with covariate selection guided by a directed acyclic graph. Clarifying the associations of these early-life conditions with muscle strength in adolescence may contribute to a better understanding of the long-term functional consequences of impaired fetal growth.

## METHOD

This longitudinal observational study was conducted using data from a population-based birth cohort carried out in São Luís, Brazil, which is part of the RPS Consortium (Consortium of Brazilian Birth Cohorts from Ribeirão Preto, Pelotas, and São Luís), developed by the Federal University of Maranhão (UFMA), the Ribeirão Preto Medical School (University of São Paulo — USP), and the Federal University of Pelotas (UFPel).^15^ This study was reported in accordance with the Strengthening the Reporting of Observational Studies in Epidemiology (STROBE) guidelines.

**Figure 1:**
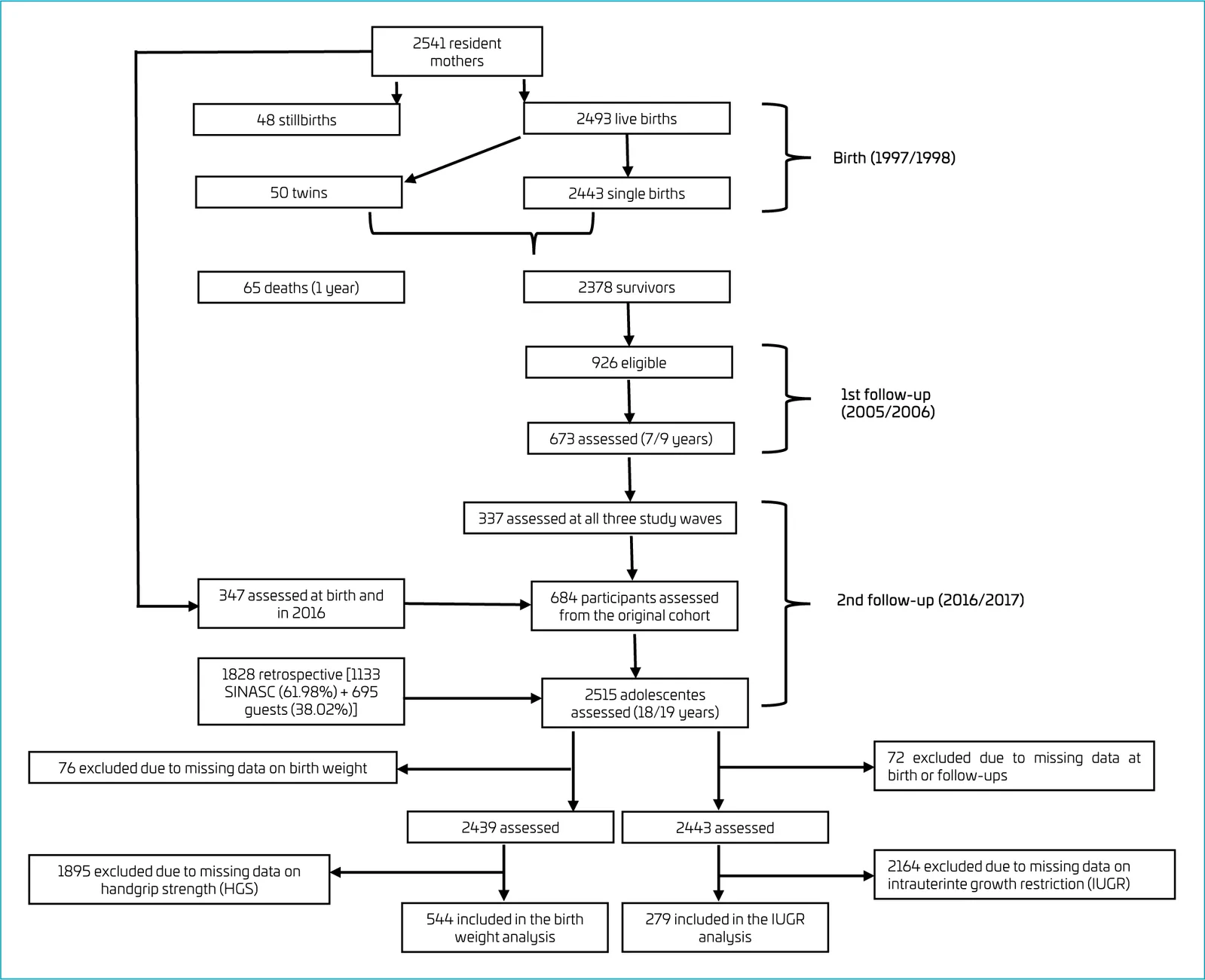
Flowchart of participants from birth to adolescence in the 1997/1998 birth cohort of São Luís, Maranhão, Brazil (1997–2017).

Source: The authors.

The initial assessment was conducted from March 1997 to February 1998 in ten maternity hospitals (public and private) in São Luís, excluding births that occurred outside hospitals and those in hospitals with fewer than 100 deliveries per year, resulting in a sample of 2493 live births.^15^ In the first follow-up (2005–2006), children with normal birth weight, LBW (<2500 g), and macrosomia (>4250 g) were invited for re-evaluation; 673 children (27% of the original cohort) were assessed at this stage. In the second follow-up, conducted during adolescence (2016–2017), 684 individuals from the original cohort (27.4%) were re-evaluated. In addition, other adolescents were retrospectively included via the Live Birth Information System (SINASC) database to increase the sample size, which had been reduced by loss to follow-up over time. The participants included in the study completed the same questionnaires as those in the original cohort, and their mothers completed a questionnaire on perinatal data. For the present study, we evaluated 544 individuals with birth weight data who participated in the baseline and second follow-up, and 279 individuals with IUGR data who participated in the first and second follow-ups. Participants with missing data for the exposures, outcome, or adjustment variables included in each model were excluded from the respective analyses, and no imputation procedures were performed (Figure 1). Data were collected using questionnaires administered by trained interviewers, with Research Electronic Data Capture (REDCap) used to record and manage the data.

To assess the outcome variable, HGS (kilogram-force — kgf), the Jamar Plus+ dynamometer from Sammons Preston was used, adjusted to the participant’s hand size. Participants were required to have their arm positioned at 90°, forearm in a neutral position, palm facing upward, seated with their feet flat on the floor, and exerting maximum force on the dynamometer.^16^ The average strength of three attempts, with a one-minute interval, was considered for the dominant hand.

Birth weight, obtained from hospital records at baseline in the participating public and private hospitals, was classified as low birth weight (<2500 g) or not (≥2500 g).^17^

To assess intrauterine growth restriction (IUGR) (yes/no), Kramer’s index^18^ was used, which divides birth weight by the estimated 50^th^ percentile weight for each gestational age, based on the reference values from the Ramos Intrauterine Growth Curve.^19^ Newborns with a value below 0.85 were classified as having IUGR. Gestational age was calculated from the date of the last menstrual period (LMP) using the following formula: date of birth minus the date of the last menstrual period divided by seven.

The following additional variables were used: self-reported maternal skin color according to the Brazilian Institute of Geography and Statistics (IBGE) (White, Black, Brown), maternal age at delivery (>20 years, 20–34 years, 35 years or older), maternal education (0–4 years, 5–9 years, 9–11 years, and 12 years or more), socioeconomic classification according to family income at birth (≤1 minimum wage, >1 to <3 minimum wages, ≥3 minimum wages), and economic class categorized as A/B, C, and D/E, with class A the wealthiest and class D/E the poorest,^20^ maternal smoking during pregnancy (yes/no), and maternal occupation (non-manual workers, unskilled manual workers, or skilled manual workers). Maternal variables were obtained in the maternity hospitals at the time of birth.

Birth variables included prematurity (yes/no), parity (one child, two children, three children, four or more children), prenatal care (yes/no), and type of delivery (vaginal or cesarean), as well as gestational age classified as preterm (delivery before 37 weeks of pregnancy), term (37–41 weeks of pregnancy), and post-term (42 weeks of pregnancy or more). Adolescent variables included years of education (0–8 years, 9–11 years, >12 years), and leisure-time physical activity assessed using an adaptation of the Self-Administered Physical Activity Checklist (SAPAC)^21^ and categorized (insufficiently active, physically active) based on the World Health Organization recommendations for ­physical activity.^22^

All statistical analyses were performed using Stata version 14.0 (StataCorp, College Station, TX, USA). Descriptive analyses were performed for all variables. Means of the outcome variable (HGS) were compared between groups using the Wilcoxon test. A chi-square test was used to compare participants from the first and second follow-ups to assess potential differences in the characteristics of the study population due to loss to follow-up.

Associations between LBW and HGS and between IUGR and HGS were estimated using crude and adjusted linear regression models, yielding the beta regression coefficient and 95% confidence interval (95%CI). Separate regression models were fitted for each exposure variable, each adjusted for its respective set of confounding variables. Because LBW and IUGR are closely related indicators of fetal growth, including both variables in the same model could introduce collinearity and misinterpretation of their associations with HGS. To examine whether the associations between the exposure variables and HGS in adolescence differed by sex, we tested interaction terms between sex and each exposure variable (IUGR and LBW) in the regression models. In contrast, the interaction between sex and LBW was not significant; therefore, sex was included only as an adjustment variable in the corresponding models.

**Figure 2:**
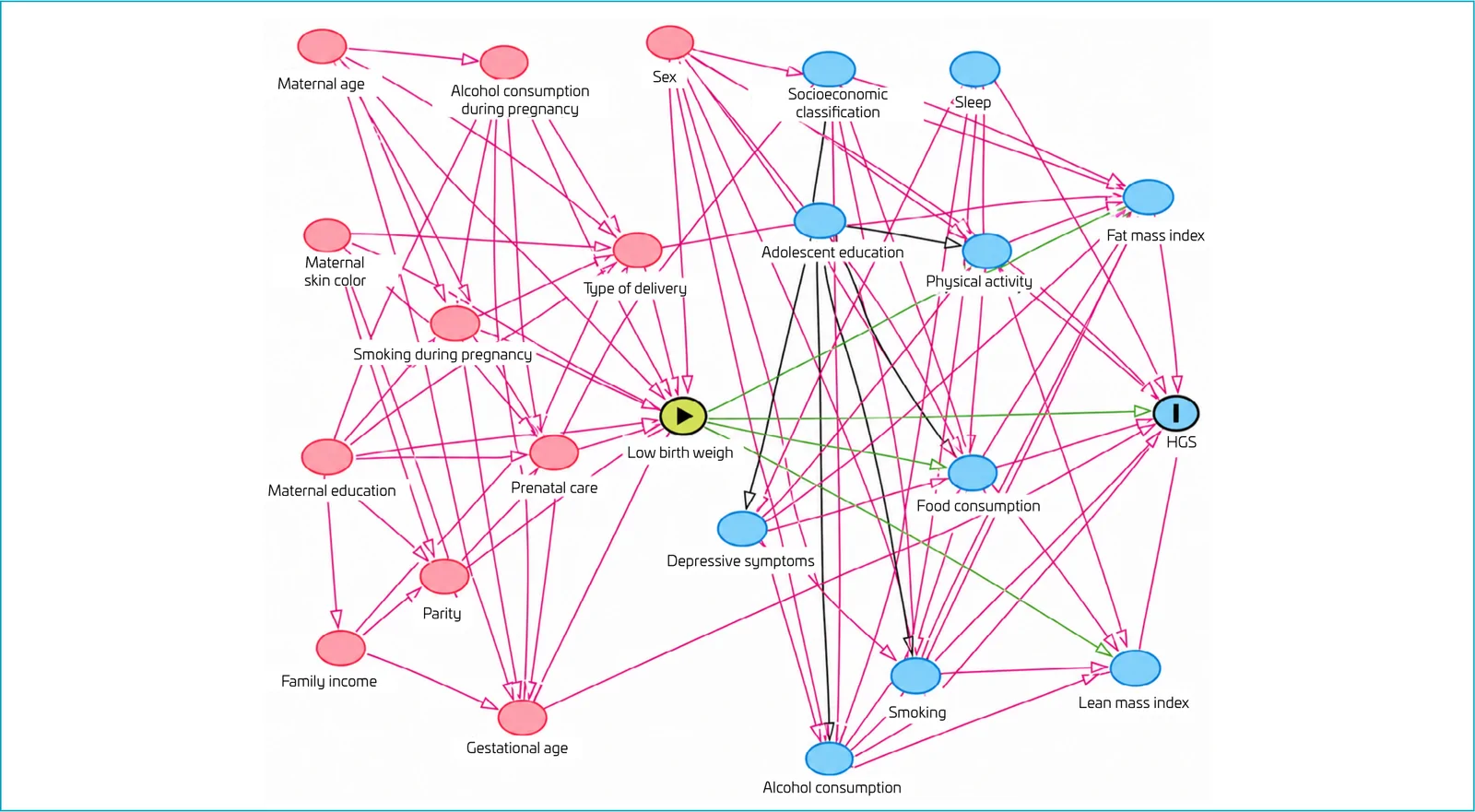
Directed Acyclic Graph of the relationship between low birth weight and handgrip strength.

Source: The authors.

**Figure 3:**
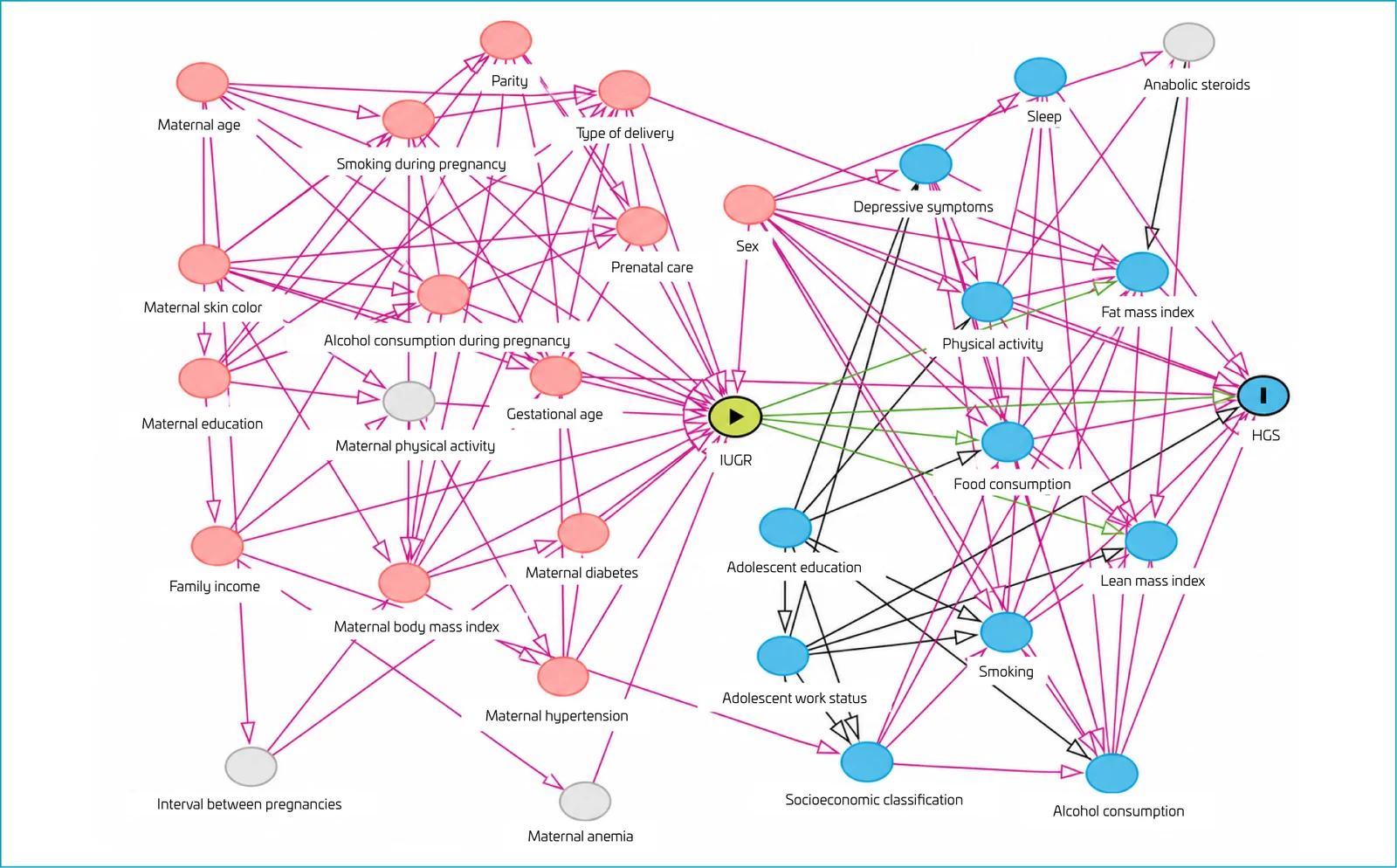
Directed Acyclic Graph of the relationship between intrauterine growth restriction and handgrip strength.

Source: The authors.

The minimum set of adjustment variables to reduce potential confounding or selection bias was determined using a Directed Acyclic Graph (DAG) created in DAGitty® software version 3.0.^23^ DAGs were employed to identify the minimal sufficient adjustment set of confounders required to estimate the total effect of the exposure variables on the outcome in the regression models. For the association between LBW and HGS, the minimum set of adjustment variables included: family income, gestational age, type of delivery, and sex. For the association between IUGR and HGS, the adjustment variables were: family income, gestational age, and type of delivery. Analyses were performed separately for the two exposure factors assessed, resulting in two figures: one for LBW and HGS (Figure 2), and another for IUGR and HGS (Figure 3).

Written informed consent was obtained from all participants at each stage of the cohort study. The study protocol was approved by the Research Ethics Committee of the University Hospital of the Federal University of Maranhão (process no. 33104-476/2005 and no. 1,302,489/20015). All procedures were conducted in accordance with the ethical principles outlined in the Declaration of Helsinki.

## RESULTS

In the distribution of participants, those included in the analysis for LBW or IUGR were compared to those excluded from the analysis. Differences were observed between those included and not included in the LBW analysis in the variables sex, type of delivery, parity, prenatal care, maternal education, and family income. Regarding participants with or without IUGR, differences were observed in birth weight, prenatal care, and maternal education (Table 1).

**Table 1:** Comparison of baseline characteristics between participants included and not included in the final analysis of the 1997/1998 Birth Cohort. São Luís (MA), Brazil, 2016.

	1997 (n=2443) Birth Weight (Birth x 3^rd^ Follow-up)			2005/2006 (n=2443) IUGR (1^st^ x 2^nd^ Follow-ups)		p-value
	Participants excluded from analysis	Participants included in analysis	p-value	Participants excluded from analysis	Participants included in analysis (n=279)	
	n (%)	n (%)		n (%)	n (%)	
Sex						
Female	904 (81.81)	201 (18.19)	<0.001	984 (89.05)	121 (10.95)	0.507
Male	995 (74.36)	343 (25.64)		1180 (88.19)	158 (11.81)	
Newborn skin color						
White	92 (59.35)	63 (40.65)	0.159	92 (59.35)	63 (40.65)	0.159
Black	21 (44.68)	26 (55.32)		21 (44.68)	26 (55.32)	
Brown	269 (58.86)	188 (41.14)		269 (58.86)	188 (41.14)	
Birth weight (grams)						
<2500	140 (75.27)	46 (24.73)	0.408	153 (82.26)	33 (17.74)	0.005
≥2500	1755 (77.90)	498 (22.10)		2007 (89.08)	246 (10.92)	
Prematurity						
Yes (<37 weeks)	241 (79.02)	64 (20.98)	0.564	268 (87.87)	37 (12.13)	0.677
Breastfeeding						
Yes	1497 (77.69)	430 (22.31)	0.463	1703 (88.38)	224 (11.62)	0.336
Type of delivery						
Vaginal	1284 (79.31)	335 (20.69)	0.009	1433 (88.51)	186 (11.49)	0.882
Cesarean	615 (74.64)	209 (25.36)		731 (88.71)	93 (11.29)	
Parity (child)						
One	912 (76.64)	278 (23.36)	0.003	1052 (88.40)	138 (11.60)	0.084
Two	564 (77.90)	160 (22.10)		638 (88.12)	86 (11.88)	
Three	231 (74.84)	78 (25.16)		269 (86.77)	41 (13.23)	
≥ Four	191 (87.21)	28 (12.79)		205 (93.61)	14 (6.39)	
Prenatal care						
Yes	1703 (76.92)	511 (23.08)	0.002	1954 (88.26)	260 (11.74)	0.041
Maternal age (years)						
<20	575 (79.97)	144 (20.03)	0.058	644 (89.57)	75 (10.43)	0.484
20–34	1237 (76.36)	383 (23.64)		1426 (88.02)	194 (11.98)	
≥ 35	85 (83.33)	17 (16.67)		92 (90.20)	10 (9.80)	
Maternal education (years)						
0–4	347 (82.62)	73 (17.38)	<0.001	382 (90.95)	38 (9.05)	0.006
5–8	829 (80.02)	207 (19.98)		917 (88.51)	119 (11.49)	
9–11	626 (72.62)	236 (27.38)		745 (86.43)	117 (13.57)	
≥12	92 (77.31)	27 (22.69)		114 (95.80)	5 (4.20)	
Family income (minimum wages)						
≤1	330 (82.29)	71 (17.71)	0.006	365 (91.02)	36 (9.98)	0.216
>1 to <3	662 (78.53)	181 (21.47)		743 (88.14)	100 (11.86)	
≥3	775 (74.81)	261 (25.19)		910 (87.84)	126 (12.16)	
Intrauterine growth restriction						
Yes	82 (65.08)	44 (34.92)	0.066	82 (65.08)	44 (34.92)	0.066

Source: The authors.

Table 2 presents the descriptive analyses in the overall sample for participants assessed at birth with the birth weight variable (n=544) and for participants assessed in the second follow-up with IUGR (n=279), stratified by sex. There were differences in prevalence by sex in maternal skin color and leisure-time physical activity. The mean HGS among adolescents was 29.26±9.38 Kgf. The mean HGS was 28.95±8.95 Kgf among participants with LBW and 29.29±9.42 Kgf among those with birth weight ≥2500 g (p=0.789). The mean HGS was 25.92±7.43 Kgf among adolescents with IUGR, and 28.28 ± 9.39 Kgf among those without IUGR (p=0.116).

**Table 2:** Descriptive characteristics of the study sample according to birth weight and intrauterine growth restriction in the 1997/1998 Birth Cohort. São Luís (MA), Brazil, 2016.

	Birth weight	IUGR		p-value
		Male	Female	
	n (%)	n (%)	n (%)	
Newborn skin color	n=277	n=277		0.069
White	63 (22.74)	22 (34.92)	41 (65.08)	
Black	26 (9.39)	16 (61.54)	10 (38.46)	
Brown	188 (67.87)	83 (44.15)	105 (55.85)	
Birth weight (grams)	n=544	n=279		0.528
<2500	46 (8.46)	16 (48.48)	17 (51.52)	
≥2500	498 (91.54)	105 (42.68)	141 (57.32)	
Prematurity	n=544	n=279		0.734
Yes	64 (11.76)	17 (45.95)	20 (54.05)	
Breastfeeding	n=513	n=264		0.622
Yes	430 (83.82)	97 (43.30)	127 (56.70)	
Type of delivery	n=544	n=279		0.267
Vaginal	335 (61.58)	85 (45.70)	101 (54.30)	
Cesarean	209 (38.42)	36 (38.71)	57 (61.29)	
Prenatal care	n=538	n=274		0.611
Yes	511 (94.98)	112 (43.08)	148 (56.92)	
Maternal age (years)	n=544	n=279		0.655
<20	144 (26.47)	34 (45.33)	41 (54.67)	
20–34	383 (70.40)	84 (43.30)	110 (56.70)	
≥35	17 (3.13)	3 (30.00)	7 (70.00)	
Family income (minimum wage)	n=513	n=262		0.454
≤1	71 (13.84)	14 (38.89)	22 (61.11)	
>1 to <3	181 (35.28)	49 (49.00)	51 (51.00)	
≥3	261 (50.88)	53 (42.06)	73 (57.94)	
Maternal smoking	n=544	n=279		0.715
Yes	27 (4.96)	5 (38.46)	8 (61.54)	
IUGR	n=279	n=279		0.525
Yes	44 (15.77)	21 (47.73)	23 (52.27)	
Economic classification	n=475	n=240		0.078
A/B	131 (27.58)	19 (32.76)	39 (67.24)	
C	240 (50.53)	52 (41.94)	72 (58.06)	
D/E	104 (21.89)	31 (53.45)	27 (46.55)	
Years of schooling (years)	n=537	n=277		0.315
0–8	40 (7.45)	8 (34.78)	15 (65.22)	
9–11	481 (89.57)	108 (43.20)	142 (56.80)	
≥12	16 (2.98)	3 (75.00)	1 (25.00)	
Leisure-time physical activity	n=544	n=279		<0.001
Insufficiently active	291 (53.49)	94 (58.02)	68 (41.98)	
Physically active	253 (46.51)	27 (23.08)	90 (76.92)	

Sample size (n) varies across variables due to missing data. IUGR: intrauterine growth restriction.

Source: The authors.

A statistically significant interaction between sex and IUGR was observed (p for interaction=0.024), whereas no interaction was found between sex and LBW (p for interaction=0.960). Therefore, analyses for IUGR were stratified by sex. Table 3 shows the crude and adjusted coefficients for the association between LBW and IUGR with HGS in adolescents. When LBW was assessed, no associations with HGS were observed. However, in the crude analysis, male adolescents who had IUGR showed lower HGS (β -4.17; 95%CI -7.67; -0.66) compared to those without IUGR. In the adjusted analysis, this association remained, with male adolescents with IUGR showing a reduction of 4.36 kgf in HGS (β -4.36; 95%CI -7.88; -0.85) compared to those without IUGR. No association between IUGR and HGS was observed among females.

**Table 3:** Crude and adjusted linear regression coefficients for the association between low birth weight and intrauterine growth restriction with handgrip strength in adolescents from the 1997/1998 Birth Cohort. São Luís (MA), Brazil, 2016.

Variables	Crude analysis	p-value	Adjusted analysis	p-value
	β (95%CI)		β (95%CI)	
Birth weight				
LBW	-0.73 (-3.55; 2.09)	0.613	0.09 (-2.15; 2.33)	0.938
IUGR				
Males	-4.17 (-7.67; -0.66)	0.020	-4.36(-7.88; -0.85)	0.015
Females	0.99 (-1.58; 3.57)	0.447	1.44 (-1.20; 34.09)	0.281

## DISCUSSION

The results showed that there was no association between LBW and HGS in adolescents. However, there was an association between IUGR and HGS in male adolescents, but not in female adolescents.

Unlike the present study, Morrison et al., in a cohort study with individuals assessed at three time points — at birth, between ages 22 and 26, and finally between 29 and 36 — showed an association between birth weight and HGS, with those born with extremely low birth weight (ELBW) having lower HGS compared to those with normal weight^6^. However, this study evaluated individuals born with ELBW (<1000g), whereas our study assessed LBW (<2500g). This difference may limit comparability and partly explain the divergent findings.

Biellemann et al.,^10^ in another cohort study with 30-year-old individuals, found an association between birth weight for gestational age and HGS in both women and men. Individuals whose birth weight was below the 10^th^ percentile for gestational age and sex, according to the Williams reference curve, were classified as small for gestational age (SGA). Additionally, birth weight for gestational age was analyzed using standardized z-score categories. Among men, those born with an appropriate weight for gestational age had higher HGS compared with those classified as SGA. Among women, those born appropriate for gestational age showed an increase of 1.2 kg of strength compared with those born SGA.^10^

Another study that also differs from the findings of the present study is the cohort study conducted with 416 Spanish children aged six to eight years, in which the authors found a positive association between birth weight and HGS in both sexes.^11^ Although that study did not specifically investigate LBW, their results indicate that higher birth weight is associated with higher HGS in children.

Although the results of the aforementioned studies contrast with those of the present study, which did not find an association between LBW and HGS in adolescents, it should be considered that the different age ranges investigated in those studies limit comparability. The scarcity of studies exploring the relationship between LBW and HGS in adolescence highlights a gap in the literature, suggesting the need for further research on this relationship in this age group.^24^ In this regard, although there is evidence that HGS in adolescence may already be determined in utero,^25^ our results indicate that other environmental, behavioral, or physiological factors, such as differences in body composition and pubertal stage, could also influence this relationship,^26^ which could explain the lack of association between LBW and HGS observed in our study.

Furthermore, Dodds et al.,^13^ in a systematic review, demonstrated that although the positive association between birth weight and muscle strength persists throughout life, the effect size is greater and more consistent between 21 and 40 years of age. It is possible that the influence of LBW on muscle strength becomes more evident at the peak of strength, which is reached in early adulthood.^13^ Considering that our study population consisted of adolescents, the association between LBW and HGS may not yet be fully expressed in this age group. In general, HGS increases progressively during adolescence, reaches its peak in early adulthood, remains relatively stable until midlife, and gradually declines thereafter.^27^

Another possible explanation may be inferred from a study by Small et al.,^28^ who assessed muscle function in children and adolescents aged 11 to 17 years born with ELBW. In that study, only dynamic muscle performance, but not static performance, was impaired among ELBW individuals, suggesting a possible neuromuscular control deficit in this group. In our study, only handgrip strength — a measure of static muscle performance — was evaluated. It is possible that, among adolescents, neuromuscular deficits associated with LBW are more evident in activities involving dynamic strength rather than in maximal isometric contractions, such as those assessed by handgrip strength.

The association between IUGR and lower HGS only in male individuals found in this paper is consistent with the findings of DiPietro and Voegtline,^29^ who describe the gestational origins of sex as one of the main moderators of human development. Their review indicates that male individuals are possibly more susceptible to unfavorable developmental outcomes because they undergo a faster growth process, resulting in greater ­vulnerability to IUGR exposure compared to females.^29^

Garay et al.^9^ assessed 124 healthy adults in a study in which birth weight adjusted for gestational age was used as a possible indicator of IUGR. Participants who were possibly exposed to IUGR had lower HGS measurements; that is, the greater the exposure to IUGR, the lower the HGS detected in these individuals. When adjusted for sex, the association was stronger among females. The authors suggest that birth size may influence muscle development, which contributes to muscle strength in adulthood and is partially mediated by lean body mass. They also note that the absence of a similar association among males in their study may be related to the smaller number of male participants, which could have limited the statistical power to detect an effect.^9^

There is evidence that the association between IUGR and lower HGS is due to alterations in utero and to the fact that growth restriction leads to a reduction in the number and/or size of muscle fibers, decreasing muscle functionality.^13^ Therefore, it is important to pay attention to the intrauterine environment, as in the early weeks of pregnancy, primary and secondary muscle fibers are formed.^10^

Moreover, chronic placental insufficiency and the resulting fetal hypoxia trigger a compensatory “brain-sparing” redistribution of blood flow, which helps maintain brain perfusion but does not fully prevent injury in vulnerable regions such as the thalamus, cerebellum, and periventricular white matter. At the cellular level, growth-restricted fetuses show increased apoptosis, reduced neurogenesis, and altered oligodendrocyte maturation, contributing to axonal injury and impaired synaptic connectivity. Consequently, studies report poorer gross and fine motor outcomes, including reduced strength, among children and young adolescents affected by IUGR.^14^ Our results show that these consequences can also persist into late adolescence.

Among the strengths of this study, we highlight its longitudinal design, which allows the establishment of a temporal sequence between exposure to the factor and the subsequent outcome. The use of validated instruments and prior training of the researchers ensured greater reliability of the data collected.

Some limitations should be noted, such as the loss of representativeness of the study sample, which is a frequent limitation in longitudinal studies. This may have occurred due to difficulties in accessing individuals from the original cohort, whether because of address changes, telephone contact issues, or refusal to continue participating in the study. The sample size was influenced by loss to follow-up, which may have introduced attrition bias. As a result, it was necessary to incorporate a retrospective cohort in order to mitigate potential selection bias. In this added sample, birth data were self-reported, which may reduce the reliability and accuracy of the information collected. Additionally, variables such as body composition and pubertal stage were not considered in the analyses. These factors may influence muscle strength during adolescence and could be explored in future studies.

The findings of this study showed that LBW was not associated with HGS in adolescents. However, IUGR was associated with lower HGS in male adolescents.

Given this, it is especially important to promote a physically active lifestyle and adequate nutrition in males who were exposed to IUGR. Activities that improve muscle strength should be encouraged from childhood, since a physically active lifestyle is established early in life.^30^

Given the evidence presented here, birth-related variables and the subsequent development of muscle strength should be further explored, as they represent a major public health concern in low- and middle-income countries.

In conclusion, LBW was not associated with HGS. However, male adolescents with IUGR had lower HGS.

## Data Availability

The database that originated the article is available upon request, with the author Rosângela Fernandes Lucena Batista (rosangela.flb@ufma.br). The data presented in this study are not publicly available due to being used under license for the current study.
